# Effects of “Football and Nutrition for Health” program on body composition, physical fitness, eating behaviours, nutritional knowledge, and psychological status among 7 to 10 years school children

**DOI:** 10.3389/fped.2023.1251053

**Published:** 2023-11-08

**Authors:** Micaela C. Morgado, Mónica Sousa, André B. Coelho, Susana Vale, Júlio A. Costa, André Seabra

**Affiliations:** ^1^ Research Centre in Physical Activity, Health and Leisure (CIAFEL), Faculty of Sport, University of Porto, Porto, Portugal; ^2^ Portugal Football School (PFS), Portuguese Football Federation (FPF), Cruz Quebrada - Dafundo, Portugal; ^3^ CIDEFES, Universidade Lusófona, Lisboa, Portugal; ^4^ CINTESIS@RISE, NOVA Medical School (NMS), Faculdade de Ciências Médicas (FCM), Universidade Nova de Lisboa, Lisboa, Portugal; ^5^ Research Center for Sport and Physical Activity (CIDAF), Faculty of Sports Science and Physical Education, University of Coimbra, Coimbra, Portugal; ^6^ Porto School of Education, Polytechnic Institute of Porto, Porto, Portugal

**Keywords:** childhood obesity, physical activity, nutrition education, recreational football, public health, noncommunicable diseases

## Abstract

**Introduction:**

Noncommunicable diseases and obesity are between the major health threat due to consumption of unhealthy foods and limited time spent on physical activities, a situation of particular concern among children. Since children spend most of their time at school, this study intends to investigate the effect of a school intervention program, which combines recreational football and nutrition education, on body composition, physical fitness, physical activity, blood pressure and heart rate, eating behaviours, nutritional knowledge, and psychological status in elementary school children.

**Methods:**

A total of 67 children, between 7 and 10 years old, were allocated into three groups: the Football Group (FG) which held 2 weekly sessions of 60 min of recreational football, the Nutrition and Football Group (NFG) which held 2 sessions per week of 60 min of recreational football plus 60 min of nutritional education and the Control Group (CG) which maintained its usual curriculum. The intervention lasted 12 weeks. All measurements were collected before and after the intervention.

**Results:**

Intervention groups significantly (*p* < 0.05) improved BMI Z-score, rest heart rate, horizontal jump and shuttle test, physical activity level, and psychosocial health. The NFG group significantly decreased (*p* < 0.05) waist-to-height ratio and blood pressure, and significantly increased (*p* < 0.05) nutritional knowledge, fruit, and fish consumption. While FG significantly decreased (*p* < 0.05) the percentage of fat mass and significantly increased (*p* < 0.05) muscle mass and performance in the 20 m sprint.

**Discussion:**

The results have shown to improve nutritional status, explosive strength, aerobic and neuromuscular fitness, as well as increase the level of physical activity. The nutritional education sessions contributed to increase nutritional knowledge and to improve the consumption of healthy food groups in a ludic-educational way. The “Football and Nutrition for Health” program was able to induce short-term improvements in several health markers, highlighting the role of the school curriculum in children's health.

## 1. Introduction

In World Health Organization (WHO) European Region, non-communicable diseases are the major health threat due to consumption of unhealthy foods, and limited time spent on physical activities, a situation of particular concern among children (1). Obesity, cardiovascular disease, and type 2 diabetes are examples of high-prevalence non-communicable diseases which are also partly interconnected (1). In this sense, prevention, and control of noncommunicable diseases have been identified in the United Nations Sustainable Development Goals as one of the key global priorities for the next decade (1).

Regarding childhood obesity, although remains a major public health problem in the WHO European Region (2, 3), rates of childhood obesity seem to be plateauing in some European countries. In Portugal overweight and obesity levels remain cautious, with one in three children in school-age are overweight (2, 3). Research from the latest round of the WHO European Childhood Obesity Surveillance Initiative (COSI) carried out in 2018–2020 indicates that 29% of children aged 7–9 years in the participating countries were found to be living with overweight (including obesity—according to WHO definitions) (2).

In recent decades, changes in dietary patterns and physical activity behaviours have been identified as likely contributors to a rise in childhood obesity (4). Physical activity is widely recognized as an important factor for prevention and treatment of childhood obesity and its comorbidities (5, 6). Although the health benefits of regular physical activity are extensively documented, 3 in 4 children and adolescents worldwide (aged 11–17 years) do not currently meet the global recommendations for physical activity (6). Another important factor that contributes to protect against overweight, obesity and other noncommunicable diseases is a healthy diet (4). COSI data has previously found that most countries indicate that childreńs level of fruit and vegetable consumption in the WHO European Region is still poor (7–9), while sugar intake is too high, with children commonly consuming more than 10% of their daily calories from added sugars (8–10).

Since children are in a period with rapid physiological and behavioural change, it is a critical time for the formation and establishment of lasting adequate nutrition, physical activity, and healthy behaviours (9). Food preferences and eating habits established in childhood and adolescence tend to be maintained into adulthood, making nutrition in childhood an important public health issue (4).

The pilot project of “‘FIFA 11 for Health’ modified program for Europe was developed in Denmark and showed positive effects on blood pressure, fat percentage, social well-being, and health knowledge (relating to physical training, healthy diet, hygiene, and mental health) (11–13). The conclusions of the studies that investigated football training in schools are encouraging. Small-sided school-based football interventions improve physical fitness (12, 14–17), heart health (15, 18–23), bone health (16, 17), psychological status (13, 24), and learning (11, 13) in children aged 8–12 years. Changes in body fat percentage and blood pressure can be achieved through dietary manipulations, physical activity interventions and other lifestyle changes, reinforcing the potential of combined interventions (12). There are several intervention programs designed to increase children's physical activity or health knowledge, but only a few programs that attempt to increase both (13, 25, 26). Furthermore, to the best of the authors’ knowledge, interventions that combine nutrition education and recreational football are unknown.

For all describe reasons mentioned above, it was created the “Football and Nutrition for Health” project, inspired in “FIFA 11 for Health” modified program for Europe, that integrates the two major determinants of health in children: physical activity and nutrition as a non-pharmacological strategy to prevent and treat lifestyle diseases. Thus, the aim of this study was to understand the effects and differences of a recreational football intervention a sole and the combined effect of nutrition education and recreational football in body composition, blood pressure, heart rate, physical fitness, eating attitudes, nutritional knowledge, and perceived psychological status, after 12-week on schoolchildren aged 7–10 years old.

## 2. Materials and methods

### 2.1. Study design and participants

The current study lasted 14 weeks: week 1 was used for the pre-tests; weeks 2 to 13 the intervention groups completed the “Football and Nutrition for Health” program, and in week 14, all groups completed post-testing with the same test battery as in week 1.

**Figure 1:**
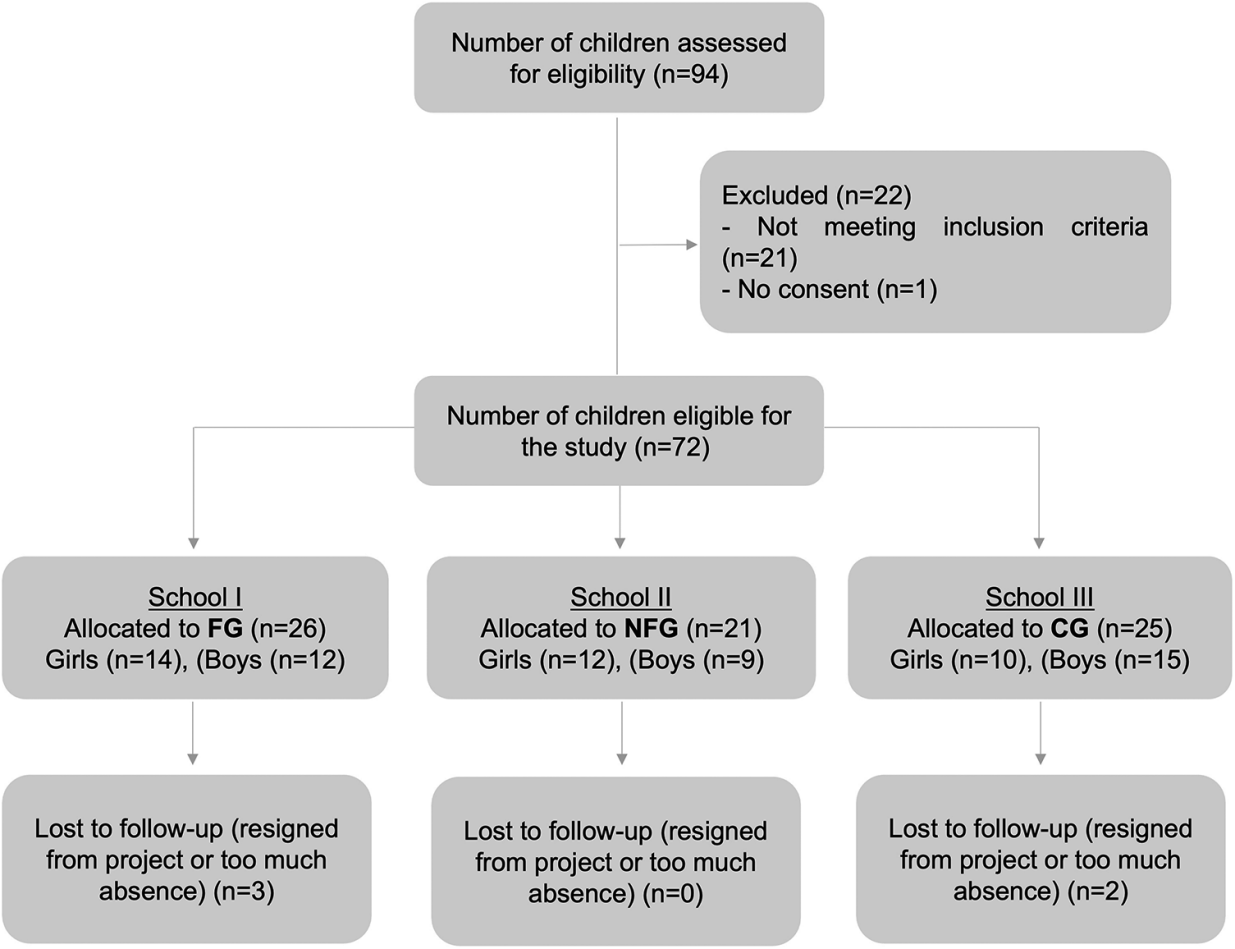
Participant flow chart of “nutrition and football for health”. FG, football group; NFG, nutrition and football group; CG, control group.

To be eligible for participation, boys and girls had to be between 7 and 10 years old and attend elementary schools in the municipality of Águeda. Children under the age of 7 were not included in the study, as well as those who were participating in any nutritional or weight loss programs. Moreover, individuals using any medication or having a pathology or clinical condition that could potentially influence the study outcomes or limit participation in physical activities were also excluded. BMI was not considered as an exclusion factor. A total of 72 Portuguese elementary school children, aged 7–10 years from three schools participated in this study and were cluster-randomized, with each school representing a cluster, into the Football Group (*n* = 26, school I), Football and Nutrition Group (*n* = 21, school II) or Control Group (*n* = 25, school III). From those, 67 children completed both pre- and post- testing. The participant selection flow chart is presented in Figure 1.

Sample size calculations were performed *a priori* for repeated measures analysis of variance (ANOVA) using the G*Power software version 3.1.9.6, considering an effect size 0.25, a statistical power of 0.95 at *p* < 0.05 (22, 27). A sample size of at least 19 in each group was required. All participants and their legal representatives were previously informed by oral and writing communication about the experimental procedures. The project was conducted in accordance with ethical procedures of the Declaration of Helsinki. The study was approved by the Ethical Committee of the Faculty of Sport of the University of Porto (nr. CEFADE 05 2019).

### 2.2. “Football and Nutrition for Health” program

In this study, the intervention groups completed the “Football and Nutrition for Health” program: Football Group (FG) performed 2 sessions per week of 60 min of recreational football and Nutrition and Football Group (NFG) performed 2 sessions per week of 60 min of recreational football plus 60 min of nutritional education, and Control Group (CG) continued their normal curriculum. It was incorporated physical activity based on recreational football practice and nutrition education in the school curriculum. The recreational football sessions were based on the structure, content, and implementation protocol for the “FIFA 11 for Health” program (13, 28) with adaptation to 12-week with 2 sessions of 60 min per week, each consisting of a play football period (i.e., teaching specific football skills, such dribbling, passing, shooting, and recreational small-sided football games). The nutrition education program consisted in a combination of six health and nutrition issues, on a weekly-basis 60 min during 12-week, based on the principles of the Portuguese food wheel rules and groups (29), consisted mostly in what are the calories, macro, and micronutrients; the nutritional traffic light label; how to prepare healthy meals; importance of fruits and vegetables; proof of the sea: choose fish, choose health.

### 2.3. Variables and measuring instruments

#### 2.3.1. Anthropometric and body composition

**Figure 2:**
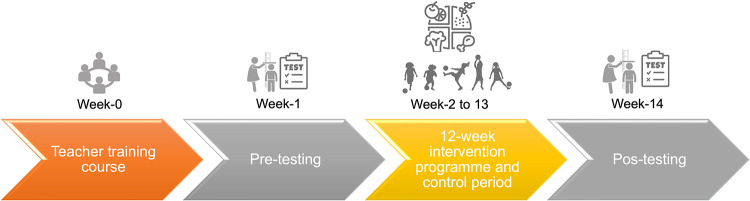
Timeline of the “Football and Nutrition for Health” program in elementary school children.

Weight and height were collected according to the ISAK protocol (30). Height was measured with a mobile stadiometer (Seca 213, Germany). BMI (kg·m^‒2^) and percentile were calculated (31). The participants were weighed bare foot in light clothes, between 9:00 and 10:00, after having breakfast before 8:00. Waist circumference was measured at the superior border of the iliac crest, a metallic tape (Holtain Ltd.), according to the protocol of the National Health and Nutrition Examination Survey (NHANES) (32). The NHANES has proposed the 90th percentile as the cut-off for identifying central adiposity. To study the central fatness, it was used waist-to-height ratios (WtHr). WtHr was calculated by dividing waist circumference (in cm) by height (in cm). The ratio between waist circumference and height was calculated. The cut-off used to represent cardiovascular risk for WtHr was 0.500 (33). Body composition was measured using the classic unifrequency electrical bioimpedance method (Akern body composition analyser, Model BIA101) to measure weight and estimate percentage body fat and lean body mass, according to protocol (34). Participants were asked to not practice physical exercise in the previous 24 h, to be preferably fasted or at least 4 h without eating and drinking (but not dehydrated), not to ingest diuretics (tea or coffee), empty bladder and bowel, during the test remove all metal (bracelets, earrings, etc.) (35) (Figure 2).

#### 2.3.2. Blood pressure and heart rate at rest

Resting blood pressure (mm Hg) was determined with the average of three measurements on the left upper arm by an automatic blood pressure monitor (36). Heart rate (bpm) was recorded using a digital blood pressure monitor (microlife BP A1 Easy, Switzerland) after subjects were seated for 10 min. (36). The average of 2 measures for systolic blood pressure (SBP) and diastolic blood pressure (DBP) was recorded. A third measurement was made if the difference between the previous two was higher than 5 mmHg. All blood pressure and heart rate measurements were conducted between 8:30 a.m. and 9:30 a.m. by the same investigator and the same automated monitor.

#### 2.3.3. Physical fitness tests

After a standardized warm-up, the children performed a physical test battery which consisted of the maximal horizontal jump length test (37), the 20 m sprint test (38) and the shuttle run test (39). To evaluate the explosive strength of the lower limbs, the maximal horizontal jump length test was conducted, where the children aimed to achieve the maximum distance in a long jump (37). For measuring the acceleration capacity and speed of the students, the 20 m sprint was performed, aiming to complete it in the shortest time possible (38). The shuttle run test was used to assess aerobic fitness, and it involved completing the maximum number of 20 m courses at a predetermined cadence signaled by a sound (39). The subjects ran in groups of up to five children to simulate a competition and ensure maximal effort.

#### 2.3.4. Physical activity monitoring

To estimate daily physical activity a tri-axial accelerometer (ActiGraph, model GT3X, Acticorp Co., Pensacola, FL, USA) was used at baseline and at the end of the study. ActiLife Software v6.13.4 was used for data processing. Before each data collection, the ActiGraph was initialized according to the manufacturer's specifications (40). Data collection by accelerometer started 1h after distribution to avoid increased physical activity (PA) results because of curiosity for device and study (40). The ActiGraph was attached to a flexible elastic belt that was fastened snugly around the waist of each child, to remain tight but not too tight. The ActiGraph was positioned on the right midaxilla line at the level of the iliac crest under or over clothing (may be in contact with the skin or over a piece of clothing). We advised that it is not visible so that other children were not tempted to touch (40). Children were asked to wear the accelerometer as soon as they got up in the morning (on waking up) and taken out at night to sleep. We also ask to only remove it for sleeping, bathing, during water-based activities and in exceptional cases like while performing contact sports such as martial arts, because of the risk of injury.

Accelerometer data files were collected in 15-s epochs according to the respectively cut point chosen to record the spontaneous and intermittent activities of children more accurately (41, 42). The accelerometers were used for 7 consecutive days and the records of physical activity performed in at least 4 days, of which 3 days a week and 1 day at the weekend, with at least 8 h of recording per day, were considered valid. Wear time validation was calculated using Troiano (43, 44) defaults and we considered days with ≥480 min of activity recordings as valid (45). Non-wear time was defined as 60 min of consecutive zeros allowing for 2 min of non-zero interruptions (46, 47). Average counts per minute (CPM) were used as a measure of total physical activity. Evenson cut-points (41), validated cut-points recommended for children, were used to estimate time spent in sedentary, light, moderate, and vigorous intensity activity in children: light (101 to ≥2,295), moderate (≥2,296 CPM), and vigorous intensity (≥4,012 CPM) physical activity (40, 42, 45, 46). The numbers of minutes per day in different intensities were determined by summing all minutes where the activity count was equal to and greater than the threshold for that intensity, divided by the number of valid days (45).

#### 2.3.5. Dietary intake and eating behaviours

##### 2.3.5.1. Dietary Intake

The collection and description of dietary intake was evaluated by a 24-hours recall using portion quantification methods with photography of home measurements (cups, bowls, and glasses) completed by the legal representatives (48). Detailed instructions were given to legal representatives to record the consumption of all foods and beverages consumed by the child, to represent the usual consumption. The instructions consist of discriminating the foods consumed, reporting the commercial name (if applicable) and the portion consumed (in weight, volume, or household measures). In the case of prepared dishes, an indication is given to provide details of the recipe, including ingredients and cooking methods. Information such as mealtime, name of meal, location of meal, and day of the week were also reported. At the end of each food record, it was asked if the registered day represents a day of usual consumption and if not, the reasons were asked. There was also an open section for comments. For nutritional data analysis, the ESHA's Food Processor Nutrition Analysis software, version 11.5 was used.

##### 2.3.5.2. Food propensity questionnaire (FPQ)

To complement the 24h recall, a food propensity questionnaire (FPQ) was applied. Parents (or the main caregiver) of children were asked to report the frequency of consumption of food and fluids items in the last month. Following the protocols proposed by IAN-AF studies (48), a food frequency questionnaire was used, including a general list of food and drink supposedly common to the different European countries (e.g., bread, rice, pasta, potatoes, fruits, red and white meat, fish, milk, butter) and a list of specific Portuguese foods and beverages important for important for assessment (e.g., certain types of vegetables, specific fresh or canned fish, certain types of cheeses). Children's nutritional attitudes and eating behaviours regarding fruit and vegetables were measured with a fruit and vegetable preference questionnaire (IAN-AF) (48). To analyse the differences between consumption frequency over the intervention, two categories were stablished according to the amount of each food group consumed. The interpretation of the data requires some care since the methodology used allows identifying the number of times a food was consumed but not the total amount ingested.

##### 2.3.5.3. Food and nutritional supplementation

The use of nutritional supplements was assessed through questions of propensity for habitual consumption, from a pre-defined list of different supplements, currently in use in the market, with the possibility of mentioning others not included in the initial list (48). The reference period to which the use of supplements refers was based on the previous month.

#### 2.3.6. Nutrition knowledge questionnaire

The questionnaire was developed with 6 questions related to the Portuguese food wheel concepts and based on the educational activities performed in the intervention program (29). The instrument was reviewed by a panel of experts for clarity of wording and instructions and then reviewed again by a panel of elementary school teachers. Statements in the questionnaire were presented in different formats: statements eliciting responses of “yes”, “no” or “do not know”; statements providing four response options (only 1 of which was correct); statements to classify foods and linking words game. Correct answers were scored with one point and incorrect and “do not know” answers were scored zero points (49–51). Each answer received a score. The scores were summed, so children received a knowledge score ranging from 0 to 20 points. Participants completed a preintervention and postintervention nutrition knowledge questionnaire to assess children's learning outcomes from the program. Higher scores indicated higher retention and understanding of the information presented in the nutritional education sessions and activities.

#### 2.3.7. Perceived psychological status

##### 2.3.7.1. Body image perception—Collins’ child figure drawing scale

Body image was analysed with Collins' child figure drawing scale (52). This pictorial instrument included seven silhouette figures of boys and girls ranging from very thin to obese. Children were asked to select the silhouette figure that best represented how their body shape currently looked (perceived) during school time. To evaluate the discrepancy between the perceived weight status (feel figure) and the actual weight status assessed by means of BMI percentile, was calculated the index FAI (feel weight status minus actual weight status inconsistency) (53). This index uses the silhouette matching technique as a proxy to confirm if there was or was not a realistic weight status perception in the subject (54). The index FAI was calculated by subtracting the feel figure assigned to the actual weight status of the participant classified as normal weight, overweight or obese based on international age- and sex-specific BMI cut-off points (55)—code 1 for underweight status (<15th); code 2 for normal weight status (15th–85th); code 3 for overweight status (85th–95th) and code 4 for obese status (95th and above)—from the code assigned to the silhouette chosen as perceived according to the following correspondence (code 1 for silhouettes 1 and 2; code 2 for silhouettes 3, 4 and 5; code 3 for silhouette 6 and code 4 for silhouette 7) (53, 56). The FAI scores range from −3 to +3: a FAI score of 0 indicates no inconsistency in weight status perception with a realistic perception of one's weight status; a negative FAI score mean that weight status is underestimated; a positive FAI score mean that weight status is overestimated (53). In the last two cases, the extent of discrepancy represents the degree of dissatisfaction in body image perception (53, 54).

##### 2.3.7.2. Pediatric quality of life inventory

To assess health-related quality of life, the adaptation of the generic scale of the Pediatric Quality Life Inventory 4.0 (PedsQL 4.0), to the Portuguese population (57) was used. It is a self-report measure with 20 items, based on a modular approach with generic and disease-specific instruments. The instructions suggest that the respondent thinks about each of the problems pointed out in terms of their occurrence during the previous month, and the answers are organized/rated on a 5-point Likert scale, ranging from 0 (never) to 4 (almost always), to indicate how much the child has problems with each area of functioning. Scoring Procedure has two steps. Step 1 is about transforming score, where items are reversed scored and linearly transformed to a 0–100 scale as follows: 0 = 100, 1 = 75, 2 = 50, 3 = 25, 4 = 0. In the step 2 the scores are calculated by dimensions, the mean score is the sum of the items over the number of items answered. If more than 50% of the items in the scale are missing, the scale scores should not be computed; If 50% or more items are completed: Impute the mean of the completed items in a scale. Three summary scores were calculated for each of the four core scales: the analysis of the results can be performed through a total score and through two sub-results: a specific one of Physical Health (6 items; corresponding to factor 1) and another one related to Psychosocial Health (emotional, social, and school functioning scales combined; 14 items; corresponding to factors 2, 3, 4). Both in the total score and in the sub-results, the values are obtained by the sum of the items divided by the number of items answered. So, total score is the sum of all the items over the number of items answered on all the Scales. The sub-result for Physical Health is the sum of the items of the physical functioning factor divided by the number of items answered. Psychosocial Health Summary Score is the sum of the items over the number of items answered in the Emotional, Social, and School Functioning Scales (57). The higher results are indicators of a better quality of life.

### 2.4. Statistical analysis

Descriptive statistics (mean values, standard deviations and percentages) were calculated for the groups at baseline and after the intervention. None of the variables analysed showed significant deviations from normal distributions (Kolmogorov-Smirnov normality test). Differences between groups at baseline and the effect of the football and nutritional intervention programmes relative to the CG was evaluated with a two-factorial repeated measures ANOVA. Percentage change (%*Δ*) between baseline and post-intervention was calculated for each variable. Effect size was calculated using eta-squared (*η*^2^). The McNemar test was used for categorical variables. Significance level was set at 0.05. Statistical analyses were conducted using SPSS version 27.0.

## 3. Results

### 3.1. Anthropometry, body composition, resting blood pressure and heart rate, physical fitness, physical activity, and perceived psychological status

Participant characteristics, anthropometry, body composition, resting blood pressure and heart rate, physical fitness, physical activity, and perceived psychological status before and after the 12-week intervention in the three groups are summarised in Table 1.

**Table 1:** Anthropometry, body composition, blood pressure and heart rate, physical fitness, physical activity, and perceived psychological status before (pre) and after (post) the 12-week intervention in the three groups.

Variables	FG (*n* = 23)			NFG (*n* = 21)			CG (*n* = 23)		
	Pre^a^	Post^a^	%*Δ*	Pre^a^	Post^a^	%Δ	Pre^a^	Post^a^	%Δ
Age (y)	8.6 ± 0.8	8.9 ± 0.8		9.4 ± 0.3	9.7 ± 0.3		8.6 ± 0.7	8.9 ± 0.8	
Anthropometry and body composition									
Height (cm)	130.2 ± 8.3**	134.5 ± 9.5***	3.3***	137.5 ± 5.2	139.3 ± 6.0	1.3	133.3 ± 8.0	136.6 ± 7.1	2.5
Weight (kg)	31.9 ± 9.2	34.2 ± 10.5	7.2	36.4 ± 8.1	36.3 ± 8.1	0.2	32.8 ± 8.0	34.5 ± 6.8	4.9
BMI	18.7 ± 3.4	18.7 ± 3.4	0.1	19.3 ± 3.6	18.7 ± 3.4	−2.7	18.3 ± 2.8	19.4 ± 2.6	5.7
BMI Z-score	0.9 ± 0.9	0.7 ± 0.9***	−21.2***	0.7 ± 0.9	0.5 ± 0.9***	−30.5***	0.7 ± 0.9	0.7 ± 0.9	−3.1
WC (cm)	66.2 ± 10.8	67.1 ± 10.0	1.3	67.4 ± 9.0	64.2 ± 8.9***	−4.3***	63.7 ± 6.6	64.5 ± 6.7	1.4
WtHr	0.50 ± 0.07	0.50 ± 0.05	−0.4	0.50 ± 0.07	0.46 ± 0.06***	−6.5***	0.47 ± 0.05	0.48 ± 0.04	2.9
Body fat (%)	28.7 ± 8.1	25.5 ± 7.7***	−11.1***	24.8 ± 8.2	24.4 ± 7.9	−1.6	26.7 ± 7.5	26.9 ± 7.4	0.9
Muscle mass (kg)	14.6 ± 3.8**	16.2 ± 4.9***	11.6***	17.9 ± 3.0	17.6 ± 2.7	−0.4	16.0 ± 3.7	15.7 ± 2.6	−0.5
Fat free mass (kg)	22.5 ± 5.8**	25.3 ± 7.5***	12.4***	26.9 ± 4.1	27.0 ± 4.0	0.3	23.8 ± 2.5	23.4 ± 3.9	−1.9
Blood pressure and heart rate									
SBP (mmHg)	100.9 ± 8.2	99.9 ± 9.8	−0.9	105.5 ± 13.5	100.8 ± 10.4***	−4.5***	102.5 ± 19.2	103.0 ± 19.4	0.5
DBP (mmHg)	61.3 ± 5.7**	60.0 ± 5.0	−2.2	68.5 ± 12.4	61.7 ± 6.6***	−9.9***	64.4 ± 9.6	64.5 ± 6.4	0.2
rHR (bpm)	89.4 ± 10.8	84.1 ± 14.9***	−5.9***	85.7 ± 12.9	78.1 ± 11.7***	−8.9***	81.5 ± 10.4	83.2 ± 7.4	2.1
Physical fitness									
Horizontal jump length (m)	1.20 ± 0.23	1.28 ± 0.25***	6.5***	1.25 ± 0.19	1.39 ± 0.22***,*	11.1***	1.21 ± 0.15	1.23 ± 0.12	1.6
20 m sprint (s)	5.00 ± 0.59**	4.85 ± 0.45**,***	−2.9***	4.64 ± 0.34	4.53 ± 0.39	−2.3	4.68 ± 0.24	4.77 ± 0.23	2
Shuttle run (m)	428.6 ± 206.6	500 ± 214.2***	16.7***	444 ± 238	642 ± 298***,*	44.1***	410 ± 162	436 ± 170	1.5
Physical activity									
Sports club active (%)	52.2	65.2	13	66.7	66.7	-	73.9	73.9	-
MVPA (min/d)	50.17 ± 15.2	58.2 ± 18.2***	15.9***	61.4 ± 19.8	68.5 ± 29.2***	12.1***	54.9 ± 14.5	55.0 ± 14.6	0.2
Psychological measures									
Index FAI	−0.6 ± 0.7	−0.4 ± 0.6***	−41***,	−0.6 ± 0.8	−0.3 ± 0.6***	−42.1***	−0.5 ± 0.7	−0.4 ± 0.7	−10.4
Physical health	70.1 ± 9.8	72.5 ± 10.5	3.6	78.8 ± 14.7	74.3 ± 15.6	−5.6	75.9 ± 16.7	75.0 ± 17.8	−1.2
Psychosocial health	70.0 ± 14.1	76.6 ± 11.6***	9.4***	68.5 ± 13.4	82.5 ± 15.7***,*	20.5***	71.2 ± 15.4	71.1 ± 14.7	−0.1
Quality of life	69.9 ± 11.5	73.8 ± 10.2	5.6	71.6 ± 12.1	76.8 ± 13.8***	7.3***	72.6 ± 14.1	72.3 ± 14.2	−0.5

FG, football group; NFG, nutrition and football group; CG, control group.

^a^ Data are presented as mean (±SD), except for the percentage of sports club active children (% of all).

* Significantly different from control at the same measuring moment (*p* < 0.05).

** Significant between-group difference (*p* < 0.05).

*** Significant baseline to post difference within the group (*p* < 0.05).

In total, 67 children (boys: 35; girls: 32) in 3 schools completed the program and both the preintervention and postintervention testing; of those, 23 were from the FG (age: 8.6 ± 0.8 years), 21 were from the NFG (age: 9.4 ± 0.3) and 23 from the CG (age: 8.6 ± 0.7 years).

No significant differences were observed at baseline between groups in weight, BMI, waist circumference, WtHr, body fat mass, systolic bloop pressure and heart rate, horizontal thrust, shuttle run test, physical activity levels, body image, physical health dimension and quality of life. Whereas significant differences were found in height [*F*_(2.64)_ = 5.53, *p* = 0.006, *η*2 = 0.15], muscle mass [*F*_(2.64)_ = 4.95, *p* = 0.010, *η*2 = 0.13] and fat-free mass [*F*_(2.64)_ = 4.24, *p* = 0.019, *η*2 = 0.12] between intervention groups at baseline. On average, height of the FG is 7.35 ± 2.22 cm lower than the heigh of the NFG, muscle mass is 3.31 ± 1.05 kg lower than NFG and the fat-free mass of the FG is 4,41 ± 1.55 kg lower than the lean body mass of the NFG. Significant differences in diastolic blood pressure were found between intervention groups at baseline [*F*_(2.64)_ = 3.16, *p* = 0.049, *η*2 = 0.09]. On average the diastolic blood pressure of the FG is 7.22 ± 2.88 mmHg lower than NFG, but there is no difference between the intervention groups and the CG. Regarding physical fitness, a statistically significant difference on 20 m sprint test [*F*_(1.64)_ = 6.01, *p* = 0.017, *η*2 = 0.14] between the intervention groups at baseline. On average, FG is 0.359 ± 0.125 s slower than NFG, but there is no difference between the intervention groups and the CG in 20 m sprint test at baseline.

Over the 12-week study period within-group improvements were observed in both intervention groups. The FG and NFG significantly decreased in BMI z-score [FG: *F*_(1.64)_ = 8.35, *p* = 0.005, *η*2 = 0.12; NFG: *F*_(1.64)_ = 8.97, *p* = 0.004, *η*2 = 0.12], and resting heart rate [FG: *F*_(1.64)_ = 6.22, *p* = 0.015,, *η*2 = 0.09; NFG: *F*_(1.64)_ = 11.81, *p* = 0.001 *η*2 = 0.16] and significantly increased in horizontal jump length [FG: *F*_(1.64)_ = 25.54, *p* < 001, *η*2 = 0.29; NFG: *F*_(1.64)_ = 74.13, *p* < 001, *η*2 = 0.54], shuttle run [FG: *F*_(1.64)_ = 11.45, *p* = 0.001, *η*2 = 0.15; NFG: *F*_(1.64)_ = 79.16, *p* < 001, *η*2 = 0.55], Index FAI [FG: *F*_(1.64)_ = 8.94, *p* = 0.004, *η*2 = 0.12; NFG: *F*_(1.64)_ = 6.80, *p* = 0.011, *η*2 = 0.10], psychosocial health dimension [FG: *F*_(1.56)_ = 6.81, *p* = 0.012, *η*2 = 0.11; NFG: *F*_(1.56)_ = 40.82, *p* < 001, *η*2 = 0.42], and moderate to vigorous physical activity (MVPA) (FG: *F*_(1.64)_ = 10.13, *p* = 0.002, *η*2 = 0.14; NFG: *F*_(1.64)_ = 7.87, *p* = 0.007, *η*2 = 0.11] over the 12-week intervention), while CG maintain constant values.

In the intervention groups, BMI z-score reduced on average 0.19 ± 0.07 scores in FG (21.2%) and 0.21 ± 0.07 scores in NFG (30.5%), resting heart rate reduced on average 5.28 ± 2.12 bpm in FG (5.9%) and 7.62 ± 2.22 bpm in NFG (8.9%), horizontal jump length improved 0.08 ± 0.02 m (6.5%) in FG and 0.14 ± 0.02 m (11.1%) in NFG, with differences between NFG and CG [*F*_(2.64)_ = 0.19, *p* = 0.016, *η*2 = 0.12], Shuttle run test improved 72 ± 21 m (16.7%) in FG and 198 ± 22 m (44.1%) in NFG, with differences between NFG and CG [*F*_(2.64)_ = 5.29, *p* = 0.007, *η*2 = 0.14], Index FAI (values closer to 0 represent consistent weight status perception) reduce 0.26 ± 0.87 scores in FG and 0.24 ± 0.91 scores in NFG, psychosocial health dimension improved on average 6.58 ± 2.42 scores in FG (9.4%) and 14.07 ± 2.20 scores in NFG (20.5%), with differences between NFG and CG [*F*_(2.56)_ = 3.42, *p* = 0.40, *η*2 = 0.11], and regarding MVPA, FG increase 8.00 ± 2.51 min/d (15.9%) the time spent in MVPA and NFG increase 7.38 ± 2.63 min/d (12.1%), with 39.1% and 57.1% of the children reaching the guideline of MVPA, respectively.

After the 12-week, the NFG significantly decreased in DBP [*F*_(1.64)_ = 14.81, *p* < 001, *η*2 = 0.19], SBP [*F*_(1.64)_ = 5.94, *p* = 0.018, *η*2 = 0.09], WC [*F*_(1.64)_ = 26.71, *p* < 001, *η*2 = 0.29], and WtHr [*F*_(1.64)_ = 20.22, *p* < 001, *η*2 = 0.24], and significantly increased in quality of life [*F*_(1.56)_ = 6.87, *p* = 0.011, *η*2 = 0.11] while the FG and CG maintain constant values between baseline and post. These differences in NFG are the result of a reduction about 6.81 ± 1.77 mmHg in DBP (9.9%), 4.71 ± 1.94 mmHg in SBP (4.5%), 2.88 ± 0.56 cm in WC (4.3%), 0.03 ± 0.01 scores in WtHr (6.5%) and an improvement around 5.22 ± 1.99 scores in quality of life (7.3%).

The FG, significantly decreased in % body fat mass [*F*_(1.64)_ = 13.31, *p* < 001, *η*2 = 0.17], significantly increased in height [*F*_(1.64)_ = 11.25, *p* = 0.001, *η*2 = 0.15], MM [*F*_(1.64)_ = 6.58, *p* = 0.013, *η*2 = 0.09], and FFM [*F*_(1.64)_ = 9.49, *p* = 0.005, *η*2 = 0.12], and significantly improved in 20 m sprint test [*F*_(1.64)_ = 6.39, *p* = 0.014, *η*2 = 0.09], while the NFG and CG maintain constant values between baseline and post. In the FG, these significant differences are the result of a reduction on average about 3.20 ± 0.88% in body fat mass percentage (11.1%), and improvements around kg 1.60 ± 1.19 kg in muscle mass (11.6%), 2.80 ± 0.96 kg in fat-free mass (12.4%), 4.29 ± 1.28 cm in height (3.3%) and 20 m sprint test by reducing on average 146 ± 58 milliseconds (2.9%), with significant differences between FG and NFG [*F*_(2.64)_ = 4.11, *p* = 0.021, *η*2 = 0.11].

In contrast, CG showed no significant changes over 12-week (*p* > 0.05).

### 3.2. Nutritional knowledge and eating behaviours

Nutritional knowledge and analysis of 24-hour recall before and after the 12-week intervention are represented in Table 2. All children consumed a minimum of five meals per day, consisting of breakfast, a morning snack, lunch, an afternoon snack, and dinner. No significant differences at baseline were observed between groups in the nutritional knowledge and eating behaviours.

**Table 2:** Analysis of 24-hour recall: daily mean nutritional intake of the participants.

	FG (*n* = 23)			NFG (*n* = 21)			CG (*n* = 23)		
	Pre^a^	Post^a^	%Δ	Pre	Post^a^	%Δ	Pre^a^	Post^a^	%Δ
NQK	10.8 ± 2.9	10.7 ± 3.4	−0.5	12.7 ± 2.5	15.3 ± 2.3*,**,***	24***	11.6 ± 2.3	11.5 ± 2.3	−0.1
24-hour recall									
Energy (kcal)	1,796.2 ± 295.2	1,751.1 ± 328.1	−1.1	1,959.9 ± 390.3	1,810.3 ± 407.1***	−6.1***	2,027.3 ± 328.6	1,998.3 ± 288.4	−1.1
Energy/Kg	55.9 ± 12.0	54.1 ± 14.2	−3.4	57.6 ± 17.5	52.7 ± 18.4***	−7.8***	65.7 ± 13.2	62.5 ± 13.0	−4.8
Protein (g)	89.0 ± 14.9	97.6 ± 13.9	11.8	99.8 ± 23.6	92.5 ± 20.0	−2.6	94.5 ± 23.4	93.9 ± 21.1	−0.1
Carbohydrate (g)	250.8 ± 66.8	202.8 ± 67.7***,*	−16.9***	252.9 ± 72.6	220.3 ± 49.1***,*	−7.9***	250.3 ± 64.5	253.5 ± 65.2	1.3
Fiber (g)	17.0 ± 7.3	14.8 ± 6.1	−10.1	16.6 ± 10.4	14.2 ± 3.9	4.5	17.9 ± 7.1	18.1 ± 7.1	1.3
Sugar (g)	93.0 ± 43.5	69.2 ± 41.0***^,^*	−18.7***	80.7 ± 30.9	71.7 ± 22.8*	−1.4	102.0 ± 40.3	98.1 ± 35.6	−2.1
Fat (g)	49.6 ± 13.7*	61.4 ± 17.5***	29.9***	62.5 ± 18.1	62.5 ± 22.9	3.9	74.7 ± 24.6	75.1 ± 24.2	0.7
Saturated fat (g)	17.0 ± 4.8*	20.3 ± 5.0***	24.1***	19.8 ± 7.8	21.7 ± 9.8	15.9	25.0 ± 11.5	25.1 ± 11.4	0.6
Sodium (mg)	1,992.8 ± 572.7	1,591.4 ± 607.5***,*	−18.0***	2,112.4 ± 744.7	1,713.3 ± 406.8***^,^*	−10.7***	2,134.3 ± 431.0	2,147.5 ± 434.5	0.6

Data are presented as mean (±SD). FG, football group; NFG, nutrition and football group; CG, control group. NKQ, Nutritional Knowledge Questionnaire.

* Significantly different from control at the same measuring moment.

** Significant between-group difference.

*** Significant within-group difference.

Between baseline and post, both intervention groups showed significant reduction in CHO intake [FG: *F*_(1.64)_ = 17.48, *p* < 001, *η*2 = 0.22; NFG: *F*_(1.64)_ = 7.39, *p* = 0.008, *η*2 = 0.10], and in sodium intake [FG: *F*_(1.64)_ = 12.89, *p* < 001, *η*2 = 0.17; NFG: F_(1.64)_ = 11.64, *p* = 0.001, *η*2 = 0.15], while CG maintain constant values. In the intervention groups, CHO intake reduced on average 47.9 ± 0.9 g in FG (16.9%) and 32.1 ± 23.6 g in NFG (7.9%), and sodium intake reduced on average 401.4 ± 34.8 mg (18%) in FG and 399.2 ± 337.9 mg (10.7%) in NFG.

Significant differences between intervention groups and control group were found in CHO [*F*_(2.64)_ = 4.02, *p* = 0.023, *η*2 = 0.11] and in sodium intake [*F*_(2.64)_ = 8.03, *p* < 001, *η*2 = 0.20] after the 12-week intervention.

Over the intervention, the NFG was the only group that significantly increase in nutritional knowledge [*F*_(1.60)_ = 47.06, *p* < 001, *η*2 = 0.44], while FG and CG maintain constant values, with between group differences [*F*_(2.60)_ = 17.05, *p* < 001, *η*2 = 0.36], and significantly decrease the total energy intake [*F*_(1.64)_ = 5.09, *p* = 0.028, *η*2 = 0.07] and consequently the energy intake/body weight [*F*_(1.64)_ = 6.51, *p* = 0.013, *η*2 = 0.09] over the intervention. On average, NFG increase 2.58 ± 0.15 scores (24%) in the nutritional knowledge and decrease 149.6 ± 16.8 Kcal in the total energy intake (6.1%) and 5.0 ± 0.9 Kcal/Kg (7.8%).

The FG increased in total fat intake [*F*_(1.64)_ = 11.47, *p* = 0.001, *η*2 = 0.15] and saturated fat intake [*F*_(1.64)_ = 8.73, *p* = 0.004, *η*2 = 0.12], and decreased sugar intake [*F*_(1.64)_ = 11.00, *p* = 0.002, *η*2 = 0.15] between baseline and post, while NFG and CG maintain constant. Differences between groups were found for sugar intake [*F*_(2.64)_ = 4.98, *p* = 0.010, *η*2 = 0.14] over the intervention. On average, FG increase 11.8 ± 3.8 g of fat intake (29.9%) and 3.3 ± 0.1 g of saturated fat (24.1%), while decreased 23.8 ± 2.5 g of sugar (18.7%).

Children's food frequency questionnaire identifies the number of times a particular food or food group was consumed during the previous month, but not the total amount ingested. The analysis of the frequency of food and drink consumption reported by the children's guardians showed that only the NFG children significantly improved the daily fruit frequency consumption [Baseline = 28.6% vs. Post = 61.9%; *χ*^2^(_1_) = 5.143; *p* = 0.016] and the frequency of fish consumption greater than or equal to 3 times a week [Baseline = 52.4% vs. Post = 85.7%; *χ*^2^(_1_) = 5.143; *p* = 0.016] over the intervention.. In addition, we observed that children from NFG tend to increase the daily consumption of vegetables [Baseline = 4.8% vs. Post = 28.6% *χ*^2^(_1_) = 3.200; *p* = 0.063] and the daily intake of soup [Baseline = 28.6% vs. Post = 57.1% *χ*^2^(_1_) = 3.125; *p* = 0.070], with no significant difference. Tables 3, 4 show the consumption frequencies in the three groups at baseline and after the intervention. Children's guardians reported not consuming any food and nutritional supplementation.

**Table 3:** Estimated differences in frequency of fruit and vegetable consumption at baseline and after 12-weeks of intervention in the three groups.

	FG (*n* = 23)			NFG (*n* = 21)			CG (*n* = 23)		
	Baseline	Post	P-value^a^	Baseline	Post	P-value^a^	Baseline	Post	P-value^a^
Fruit (%)									
≤6 times/week	26.1	39.1	0.375	71.4	38.1	0.016b^b^	39.1	47.8	0.500
Daily	73.9	60.9		28.6	61.9		60.9	52.2	
100% fruit juice (%)									
≤6 times/week	87.0	100.0	0.250	81.0	85.7	1.000	91.3	91.3	1.000
Daily	13.0	0		19.0	14.3		8.7	8.7	
Soup (%)									
≤6 times/week	26.1	30.4	1.000	71.4	42.9	0.070	34.8	43.5	0.727
Daily	73.9	69.6		28.6	57.1		65.2	56.5	
Vegetable (%)									
≤6 times/week	52.2	56.5	1.000	95.2	71.4	0.063	65.2	73.9	0.625
Daily	47.8	43.5		4.8	28.6		34.8	26.1	

Data are presented as percentage.

^a^ *χ*^2^ test (McNemar); FG, football group; NFG, nutrition and football group; CG, control group.

^b^ Significant within-group difference.

**Table 4:** Estimated differences in frequency of food and drink consumption at baseline and after 12-weeks of intervention in the three groups.

	FG (*n* = 23)			NFG (*n* = 21)			CG (*n* = 23)		
	Baseline	Post	P-value^a^	Baseline	Post	P-value^a^	Baseline	Post	P-value^a^
Bread (%)									
<3 times/week	13.0	8.7	1.000	4.8	9.5	1.000	17.4	8.7	0.500
≥3 times/week	87.0	91.3		95.2	90.5		82.6	91.3	
Cereal (%)									
<3 times/week	30.4	47.8	0.219	42.9	42.9	1.000	47.8	47.8	1.000
≥3 times/week	69.6	52.2		57.1	57.1		52.2	52.2	
Cookies (%)									
<3 times/week	39.1	43.5	1.000	19.0	38.1	0.219	34.8	39.1	1.000
≥3 times/week	60.9	56.5		81.0	61.9		65.2	60.9	
Fish (%)									
<3 times/week	17.4	26.1	0.625	47.6	14.3	0.016^b^	47.8	56.5	0.688
≥3 times/week	82.6	73.9		52.4	85.7		52.2	43.5	
Meat (%)									
<3 times/week	8.7	21.7	0.375	14.3	14.3	1.000	21.7	21.7	1.000
≥3 times/week	91.3	78.3		85.7	85.7		78.3	78.3	
Eggs (%)									
<3 times/week	56.5	43.5	0.453	47.6	57.1	0.688	52.2	52.2	1.000
≥3 times/week	43.5	56.5		52.4	42.9		47.8	47.8	
Milk (%)									
<3 times/week	0.0	4.3	1.000	9.5	14.3	1.000	30.4	30.4	1.000
≥3 times/week	100.0	95.7		90.5	85.7		69.6	69.6	
Cheese (%)									
<3 times/week	47.8	30.4	0.219	42.9	42.9	1.000	56.5	56.5	1.000
≥3 times/week	52.2	69.6		57.1	57.1		43.5	43.5	
Yogurt (%)									
<3 times/week	4.3	26.1	0.063	19.0	33.3	0.250	30.4	30.4	1.000
≥3 times/week	95.7	73.9		81.0	66.7		69.6	69.6	
Sweet snacks (%)									
<3 times/week	78.3	82.6	1.000	57.1	76.2	0.125	65.2	65.2	1.000
≥3 times/week	21.7	17.4		42.9	23.8		34.8	34.8	
Salty snacks (%)									
<3 times/week	100.0	95.7	1.000	85.7	95.2	0.500	87.0	91.3	1.000
≥3 times/week	0.0	4.3		14.3	4.8		13.0	8.7	
Soft drinks (%)									
<3 times/week	87.0	82.6	1.000	71.4	66.7	1.000	69.6	73.9	1.000
≥3 times/week	13.0	17.4		28.6	33.3		30.4	26.1	

Data are presented as percentage.

^a^ *χ*^2^ test (McNemar); FG, football group; NFG, nutrition and football group; CG, control group.

^b^ Significant within-group difference.

## 4. Discussion

To the best of our knowledge, this study is the first to examine the effects of a 12-week football and nutrition intervention program on several health markers, nutritional status, and fitness profiles.

The significant differences observed at baseline in muscle mass and fat-free mass between the intervention groups suggest that NFG was more active than FG before the intervention. Furthermore, differences in body composition, may be associated with an advanced maturity in the children.

The present findings showed a significant reduction in BMI z-score in the FG and NFG children, in accordance with a 6-month intervention with obese boys (22). Previous interventions focused on school-based physical activity have shown potential for yielding positive effects on body composition. Specifically, these interventions have led to reductions in BMI (58) and slight improvements in BMI z-scores (59).

The significantly increase of muscle mass and fat free mass in FG, with a greater change score, 11.6% and 13% respectively, is similar to reported in previous studies (12, 15, 22). The increase in muscle mass could additionally indicate muscle hypertrophy as a result of additional exercise (15, 17). A possible explanation for muscle mass in NFG does not significantly increase over the intervention is that this group started the intervention with a significant greater muscle mass contribution than FG.

In the present study, only the FG experienced significant reductions in body fat mass percentage, with no differences between groups. These findings are in accordance with other school-based physical activity interventions with 11-week (12, 15, 60), 12-week (23), and 6-month intervention (22). The reduction in the percentage of body fat mass in the NFG was not significant after the intervention, possibly because the participants started the study with a lower percentage compared to the other groups.

Nevertheless, we found a positive influence of school-based physical intervention on modulating the central-adiposity markers of the NFG, the only group that significantly decreased waist circumference (−4.2%) and WtHr (−6.2%), specific markers of upper body fat accumulation in children and able to predict cardiovascular disease risk factors (33, 61, 62). These findings concur with a 12-week recreational football program with obese adolescents (23), and with a 6-month intervention with obese boys (18, 22). In the present study, 56.8% of the intervention participants had a mean value of WtHr greater than 0.500 at baseline, meaning this population should be considered as at high-risk of developing cardiometabolic comorbidities (33). The decrease in waist circumference and WtHr verified in the NFG participants demonstrates that school-based physical intervention with recreational football and nutritional education contributes to reducing the risk of developing cardiovascular comorbidities. WtHr is more closely linked to childhood morbidity than BMI and it should be used as an additional or alternative measure to BMI in children as well as adults (33).

Paediatric obesity and lifestyle patterns have an important influence on the overall risk of cardiovascular diseases (63) and the prevalence of paediatric hypertension has been observed to rise concurrently with the increase in childhood obesity rates (18, 64). In the present investigation, both intervention groups improved their resting heart rate. Notably, the NFG significantly improved both DBP and SBP. These findings highlight the positive impact of the program on blood pressure, aligning with prior research that reinforces the favourable effects of physical activity interventions in reducing systolic and diastolic blood pressure (65).

The observed improvements concerning DBP and SBP in the NFG are similar to or even larger than reported in other comparable football intervention studies with 11 weeks (12, 15, 20). In a 12-week recreational football program with obese adolescents, significant reductions in SBP were found, but no changes were found for DBP (23). In contrast, in a 10-week football training intervention for school children aged 9–10 years no changes were observed in none of the cardiovascular parameters (20).

For adults, the risk of cardiovascular mortality and morbidity is reduced by 13% for every 5 mm Hg reduction in SBP (66). Although no equivalent risk calculations have been made in children, a reduction in blood pressure of this magnitude is associated with less arterial stiffness and a decrease in the rate of progression of atherosclerosis in adulthood (12, 67, 68). The significant reduction of 4.7 mmHg in SBP that we found in NFG is a good indicator that a multidisciplinary program with physical activity and nutritional education could reduce the risk of cardiovascular diseases.

Children with higher blood pressure are more likely to become hypertensive adults, and the observed decrease in resting blood pressure and resting heart rate in the intervention groups is an important finding, as in normotensive children, no clear link has been established between physical activity and blood pressure (21, 63). These results suggest that physical activity programs inserted in a school environment are feasible to reduce blood pressure in normotensive children, and the potential of such interventions for the primary prevention of hypertension and cardiovascular diseases clearly warrants further study.

Cardiovascular disease risk factors have been demonstrated to be associated with children level of physical activity and was highly associated with a lower level of fitness (69). Moreover, it is important to note that interventions focusing on physical activity within a school-based setting can potentially enhance both physical activity and fitness among healthy young individuals (59, 70).

Over the “Football and Nutrition for Health” program, both intervention groups improved horizontal jump length and shuttle run test performance during the program with no differences between intervention groups. Improvements in explosive strength in the transition from childhood to adolescence are associated with positive changes in bone mineral density (71). Additionally, and more generally, explosive strength is inversely related to risk factors for cardiometabolic diseases (71). This result in horizontal jump length performance is similar to 11 weeks intervention (15), to 6-month small-sided football intervention (72), and to 10-month intervention of small- sided games (17). On the other hand, studies with 8 weeks of football training sessions found that is not sufficient to result in between-group differences (14) and with a lasting 11–12 weeks (12, 60, 73) found no significant effects on horizontal jump length performance.

Shuttle run test improved in both intervention groups, revealing a better aerobic fitness after the 12-week intervention, being associated with a lower risk of cardiometabolic diseases, obesity, diabetes, and other health problems during the entire life cycle (74). Other studies have reported similar effects of recreational football programs on aerobic fitness performance in children after 6 weeks (16, 75), 11 weeks (12, 15) and 10 months (17). The high-intensity football training is associated with high-intensity intermittent aerobic exercise capacity and can be effective in improving aerobic fitness (11, 15, 19, 76).

Both intervention groups improved the performance in the 20 m sprint test, but only the FG had a significant difference between baseline and post intervention. Although, on average, the NFG was faster (4.53″) than FG (4.85″) to complete the 20 m sprint test in the post-intervention, the FG reduced they time in 2.5%, a good indicator of bone tissue health and is inversely related to risk factors for cardiometabolic diseases (77). Improvements in the 20 m sprint test are in accordance with a study with modified “FIFA 11 for Health” program for non-communicable diseases (12). On the other hand, a study with 8 weeks of football training sessions was not sufficient to result in between-group differences in sprint (14).

The most recent results from Portugal's 2021 Report Card on Physical activity for Children and Adolescents shows that less than 30% of children and adolescents achieve physical activity guidelines (78). In our study, both intervention groups improved the moderate to vigorous physical activity (MVPA) from baseline to post intervention, approaching or reaching the WHO recommendations for children (5). In a 6-month studies with overweight children, no differences in the average minutes of MVPA per day were found (18, 22).

Prior research indicates that engagement in physical activity has a noteworthy positive impact on the moods of children and adolescents (79). Concerning the psychological status, the present intervention was effective in improving scores for the sub-result of psychosocial health status (emotional, social, and school functioning dimensions) in FG and NFG. These findings provide additional support for the previously reported benefits of physical activity programs in promoting the well-being of children (11, 13, 80).

In a comparable study, scores for the PedsQL questionnaire also identified significant improvements in the social and school dimension of well-being for the intervention group (13). Likewise, other study examining the effects of a comparable program on well-being demonstrated positive enhancements in both physical and psychological well-being (80). Moreover, in previous programs with obese boys was observed an improvement on perceived psychological status, showing that recreational football as a team-based activity has the potential to promote teamwork, sharing and social interactions that offer opportunities to improve perceived psychological status (22, 81, 82).

Intervention groups also showed an improve in the Index FAI score, revealing a more consistent body image and weight status perception. Children with overweight were more likely to underestimate their actual weight status and in obesity prevention such underestimation may be a barrier for behavioural change (83). Our results are in accordance with previous 5-month (24) and a 6-months intervention programs based on the practice of football (22, 82).

The positive influence of the “Football and Nutrition for Health” program on children's perceived psychological status underscores the significance of school-based initiatives in promoting mental health. It aligns with the goals outlined in the European Mental Health Action Plan, which advocates for impactful measures to enhance mental health and well-being. Specifically, this involves implementing comprehensive mental health promotion programs within schools (84).

The implementation of interdisciplinary and playful educational health interventions within school environments has been shown to enhance knowledge of topics related to eating and nutrition. This approach proves effective in promoting healthier habits among children, as it fosters an improved understanding of dietary concepts through comprehensive education (26, 85).

Differences between the baseline and post-intervention nutritional knowledge questionnaire in the NFG revealed significant increases in children's nutritional knowledge and significant differences between groups. NFG improved the nutritional knowledge in 2.6 scores (24%) after the 12 sessions. Likewise, in a 9-weeks school-based intervention the mean total nutrition knowledge increase by 1.1 scores (49). In a 5-month educational health intervention increased children's knowledge of eating and nutrition (26). In addition, the Program Obesity Zero, revealed that the children's knowledge concerning healthy diet, increased (5.8 scale point) after 6 months (A. Rito et al., 2012). Finally, in MUN-SI, a study about the Mediterranean Diet knowledge, was observed values between +12.1% to 21.1%, between 2016 and 2019 (86).

Dietary records revealed differences in the total energy intake and in the ratio kcal/body weight from the baseline to post-intervention in NFG. Children from NFG decreased carbohydrate, and sodium intake. Children from FG decreased carbohydrate, sugar, and sodium intake, but increased total fat, and saturated fat. These changes observed in the intake of carbohydrates, fats and sodium remained within the reference values (87, 88). Although sugar intake was reduced in the FG, it remained above the recommendations (87).

Surprisingly, the FPQ revealed that children from NFG had a low frequency of fruit, soup and vegetable consumption in the pre intervention compared with FG and CG. After realizing that the school is the environment where this consumption is most frequent (through the lunch meal), we noticed a difference in relation to other schools: the lack of a supervisor who encourages the consumption of these foods. At the end, the FPQ reported by the guardians, revealed an increase of fruit similar to FG frequency consumption. We believe that nutrition education classes, focusing on the role of fruits and vegetables in children's health and growth, influenced their consumption after the intervention.

Similarly, MUN-SI program showed a positive behavioural change in the preference for including fruit and vegetables in home brought to school lunches (+2.3% in 2015/2016) and at breakfast (+16% in 2014/2015 and +11.9% in 2017/2018) (86). In Program Obesity Zero, after 6 months, there was also an improvement in children's attitudes regarding healthy foods such as brown bread, fruits and vegetables, fish, milk, and yoghurt (89). In “CHILDREN study”, after a 12-month school-based nutrition and physical activity intervention program the intervention group also revealed higher daily consumption of fruits (90).

Moreover, we speculate that the School Fruit Scheme (91), a national strategy for the supply of fruit implemented since 2009, associated with positive messages from nutrition educational classes could encourage the consumption of fruit among children. To school-based programs, we suggest combined nutritional education sessions (through positive messages and ludic environment) with programs that offers healthy food consumption in order to achieve a positive impact on eating behaviour.

In summary, the program revealed improvements in horizontal jump length, shuttle run performance, and MVPA, induced multiple effects on cardiorespiratory health, promoted a consistent body image and weight status perception (index FAI), and improved the psychosocial health status after 12-week. Furthermore, the intervention resulted in muscle mass and fat-free mass increases and reductions in BMI z-score, body fat mass percentage and rHR in the FG. While NFG decreased BMI z-score, waist circumference and WtHr, it improved DBP, SBP and rHR, increased fruit daily consumption and revealed an increase in nutritional knowledge. The “Football and Nutrition for Health" program confirmed the hypothesis that a combination of recreational football training and nutritional education classes are capable to induce short-term improvements in body composition, physical fitness, physical activity, eating habits, nutritional knowledge, and psychological status among children aged 7–10 years.

## 5. Strengths and limitations

The present study has limitations that should be acknowledged. The children were not randomly assigned to their respective groups. Furthermore, the dietary intake data relied on dietary records reported by parents, which may result in under-report or over-report of habitual nutritional intakes or changes in nutritional-intake patterns. Nevertheless, dietary records are widely used methods for the assessment of dietary intake valid in child populations. On the other hand, it is important to acknowledge the strengths of this study. It contributes to the existing knowledge by exploring the potential of a school-based intervention involving nutrition education and football, which, to the best of our knowledge, has not been previously undertaken. Moreover, the three groups were similar at baseline in the major characteristics. The utilization of accelerometers, an objective measure, further strengthened the design of our study. Overall, these strengths contribute to the robustness and credibility of our study's findings.

## 6. Conclusion

This study highlights the role of the school-based programs as an important determinant of physical activity levels in elementary schoolchildren. Introducing nutritional education and recreational football in elementary schools could be a great and effective strategy to improve physical activity, physical fitness, body composition, nutritional habits, nutritional knowledge and to prevent noncommunicable diseases in boys and girls, constituting an effective tool in health education and promotion. The outcomes of this study hold considerable importance and interest for a diverse audience, encompassing parents, educators, teachers, school administrators, and policymakers. These results strongly advocate for the inclusion of active school programs within curricula, thereby fostering the promotion of children's health. These data reinforce and justify that priority should be given to the development of national action programs that encourage the adoption of healthier lifestyles and to the creation of structural and environmental conditions favourable to child health. Further studies are needed to assess whether this intervention can form part of a more complex behaviour change intervention to prevent childhood obesity.

## Data Availability

The original contributions presented in the study are included in the article/Supplementary Material. Further inquiries can be directed to the corresponding author.
